# UNCOVERING NOVEL EPIGENOMIC SIGNATURES IN HPV AND NON-HPV HEAD AND NECK SQUAMOUS CELL CARCINOMA

**DOI:** 10.51894/001c.123033

**Published:** 2024-08-30

**Authors:** Christian Gluck

**Affiliations:** 1 Otolaryngology Detroit Medical Center

## 59

### INTRODUCTION

The molecular mechanisms driving the heterogeneity that exists in HNSCC is still poorly understood. Identifying the master regulators of this heterogeneity will help uncover key oncogenic pathways critical to the tumorigenicity of HNSCC and help uncover novel targeted therapy.

### OBJECTIVES

1. To define HNSCC heterogeneity by mapping epigenetic and transcriptomic profiles in HPV and Non-HPV HNSCC that highlight crucial and unique pathways and biomarkers that define each subtype of head and neck cancer. 2. To identify the master regulator transcription factors that control the defined HPV and Non-HPV cis-regulatory networks. 3. To predict novel therapeutic regimens by leveraging the subtype specific network analysis.

### METHODS

A panel of 14 different HNSCC cell lines, which represent HPV and non-HPV tumors, underwent comprehensive transcriptomic and epigenetic profiling to undercover the key oncogenic pathways specific of each subtype. Using bioinformatics driven analysis of the gene expression and regulatory networks, specific master regulators were identified that drive HPV and non-HPV tumorigenicity. These signatures were validated in corresponding human HNSCC and additionally, predicted novel therapeutic regimens were identified to target these regulators.

### RESULTS

Crucial subtype-specific cis-regulatory networks, which comprise of enhancers, the transcription factors that bind them and the activated oncogenic pathways, were uncovered in HPV and non-HPV HNSCC. Activated networks driven by TP63, E2F the ETS family of oncogenic transcription factors were identified and subsequently used to predict novel targeted therapies that were validated in cell line models. These analyses led to identification of promising new potential treatments that can be leveraged to address the heterogeneity of HNSCC.

### DISCUSSION/CONCLUSIONS

Master oncogenic transcription factors and the oncogenic networks they regulate offer us new avenues of targeted therapies in HPV and non-HPV HNSCC. From our integrated genomic and epigenomic data, we have identified potential small molecules that can both directly and indirectly target oncogenic transcription factors and the molecular machinery the underlie the epigenetic state of HNSCC cancer subtypes. These results from preclinical models, if validated in HNSCC patient tumor samples can be leveraged for personalized cancer treatments.

